# Supplementary material for: Impact of community piped water coverage on re-infection with urogenital schistosomiasis in rural South Africa
Source: eLife. 2020 Mar 17;9:e54012. doi: 10.7554/eLife.54012 (PMC7108860; doi:10.7554/eLife.54012)
Supplement: Source data 1. [file elife-54012-data1.docx]

**Source data 1: Prevalence of re-infection, intensity of re-infection and re-infection rate (per 100-person year of follow-up) among individuals treated at baseline for *S. haematobium* infection*.***

| Covariate | Re-infected  **% (n/N)** | Rate of re-infection **(95% CI)** | Light re-infection  **% (95% CI)**  **N=55** | Heavy re-infection  **% (95%CI)**  **N=30** |
| --- | --- | --- | --- | --- |
| Overall | 22.5 (85/378) | 17 (10- 25) | 14.6 (11.2- 18.5) | 7.9 (5.4- 11.1) |
| **Gender** |  |  |  |  |
| Female | 17.7 (21/119) | 8 (2- 24) | 11.8 (6.6- 18.9) | 5.9 (2.4- 11.7) |
| Male | 24.7 (64/259) | 20 (12- 32) | 15.8 (11.6- 20.9) | 8.9 (5.7- 13.0) |
| **Baseline age** |  |  |  |  |
| ≤10 | 29.0 (20/69) | 13 (3- 37) | 15.9 (8.2- 26.7) | 13.0 (6.1- 23.3) |
| 11 | 19.1 (20/105) | 20 (8- 41) | 13.3 (7.5- 21.4) | 5.7 (2.1- 12.0) |
| 12 | 23.3 (24/103) | 21 (8- 46) | 15.5 (9.1- 24.0) | 7.8 (3.4- 14.7) |
| ≥13 | 20.8 (21/101) | 12 (4- 29) | 13.9 (7.8- 22.2) | 6.9 (2.8- 13.8) |
| **Community piped water (quartiles)** |  |  |  |  |
| (lowest) 1 | 18.8 (15/80) | 16 (6- 34) | 10.0 (4.4- 18.8) | 8.8 (3.6- 17.2) |
| 2 | 18.8 (15/80) | 23 (8- 50) | 11.3 (5.3- 20.3) | 7.5 (2.8- 15.6) |
| 3 | 14.1 (11/78) | 10 (2- 29) | 10.2 (4.5- 19.2) | 3.9 (0.8- 10.8) |
| 4 | 18.8(16/85) | 19 (7- 42) | 12.9 (6.6- 22.0) | 5.9 (1.9- 13.2) |
| **Household access to water (2007)** |  |  |  |  |
| No piped water | 8.5 (4/47) | 13 (3- 39) | 6.4 (1.3- 17.5) | 2.1 (0.1- 11.3) |
| Piped water | 24.5 (81/331) | 17 (10- 27) | 15.7 (12.0- 20.1) | 8.8 (5.9- 12.3) |
| **Household assets quintiles** |  |  |  |  |
| (poorest) 1 | 15.3 (13/85) | 9 (2- 26) | 9.4 (4.2- 17.7) | 5.9 (1.9- 13.2) |
| 2 | 22.1 (17/77) | 10 (2- 31) | 15.6 (8.3- 25.6) | 6.5 (2.1- 14.5) |
| 3 | 20.6 (15/73) | 9 (1- 31) | 16.4 (8.8- 27.0) | 4.1 (0.9- 11.5) |
| 4 | 26.8 (19/71) | 39 (16- 76) | 14.1 (7.0- 24.4) | 12.7 (6.0- 22.7) |
| 5 | 30.0 (18/60) | 30 (10- 69) | 18.3 (9.5- 30.4) | 11.7 (4.8- 22.6) |
| **School grade** |  |  |  |  |
| Grade 5 | 22.9 (50/218) | 15 (7- 26) | 14.2 (9.9- 19.6) | 8.7 (5.3- 13.3) |
| Grade 6 | 21.9 (35/160) | 19 (9- 36) | 15.0 (9.9- 21.5) | 6.9 (3.5- 12.0) |
| **Altitude (meters)** |  |  |  |  |
| < 50 | 35.5 (11/31) | 0 (0- 36) | 25.8 (11.9- 44.6) | 9.7 (92.0- 25.8) |
| 50-100 | 20.8 (42/202) | 22 (12- 37) | 12.4 (8.2- 17.7( | 8.4 (5.0- 13.10 |
| 100-150 | 20.6 (26/126) | 10 (3- 25) | 12.7 (7.4- 19.8) | 7.9 (3.9- 14.1) |
| 150-200 | 20.0 (2/10) | 0 (0- 62) | 20.0 (2.5- 55.6) | 0 (0- 30.8) |
| ≥200 | 44.4 (4/9) | 79 (10- 290) | 44.4 (13.7- 78.8) | 0 (0- 33.6) |
| **Distance to water body** |  |  |  |  |
| <1 km | 22.5 (31/138) | 21 (9- 39) | 9.4 (5.1- 15.6) | 13.0 (7.9- 19.8) |
| 1-2 km | 23.6 (33/140) | 16 (6- 32) | 17.9 (11.9- 25.2) | 5.7 (2.5- 10.9) |
| 2-3 km | 25.0 (18/72) | 18 (5- 45) | 20.8 (12.2- 32.0) | 4.2 (0.9- 11.7) |
| >3 km | 10.7 (3/28) | 7 (0.0- 37) | 7.1 (0.9- 23.5) | 3.6 (0.1- 18.3) |
| **Toilet in household** |  |  |  |  |
| No toilet | 20.0(12/70) | 17 (6- 40) | 12.9 (6.1- 23.0) | 7.1 (2.4- 15.9) |
| Toilet | 23.1(71/308) | 16 (9- 27) | 14.9 (11.1- 19.4) | 8.1 (5.3- 11.7) |
| **Land cover classifications** |  |  |  |  |
| Sparse shrubland | 28.3 (17/60) | 26 (7- 67) | 15.0 (7.1- 26.6) | 13.3 (5.9- 24.6) |
| Closed shrubland | 22.4 (47/210) | 14 (7- 25) | 14.8 (10.3- 20.3) | 7.6 (4.4- 12.1) |
| Open shrubland | 21.5 (20/93) | 22 (9- 45) | 15.1 (8.5- 24.0) | 6.5 (2.4- 13.5) |
| Thicket | 6.7 (1/14) | 0.0 (0.0- 50) | 6.7 (0.2- 31.9) | 0.0 (0.0- 21.8) |
