# Supplementary material for: Impact of community piped water coverage on re-infection with urogenital schistosomiasis in rural South Africa
Source: eLife. 2020 Mar 17;9:e54012. doi: 10.7554/eLife.54012 (PMC7108860; doi:10.7554/eLife.54012)
Supplement: Supplementary file 1. — Model 1 presents results from a univariable negative binomial model and Model 2 presents results from a multivariable negative binomial model (N = 253). [file elife-54012-supp1.docx]

**Supplementary File 1: Predictors of *S. haematobium* re-infection using data from the first follow up round only.** Model 1 presents results from a univariable negative binomial model and Model 2 presents results from a multivariable negative binomial model (N=253).

|  | Model 1: Univariable analysis  (N=253) | | | Model 2: Multivariable analysis  (N=253) | | |
| --- | --- | --- | --- | --- | --- | --- |
| **Covariates** | **IRR** | **95%CI** | **P-value** | **aIRR** | **95%CI** | **P-value** |
|  |  |  |  |  |  |  |
| **Female (**ref: Male**)** | 0.16 | (0.04- 0.64) | 0.009 | 0.20 | (0.08- 0.50) | 0.001 |
|  |  |  |  |  |  |  |
| **community piped water coverage** | 0.96 | (0.93- 0.99) | 0.014 | 0.97 | (0.95- 1.00) | 0.055 |
|  |  |  |  |  |  |  |
| **Age at testing** | 0.56 | (0.35- 0.91) | 0.020 | 0.70 | (0.50-0.97) | 0.032 |
| **Altitude Class (**ref: <100 m**)** |  |  |  |  |  |  |
| 100 - 150 m | 0.21 | (0.06-0.83) | 0.025 | 0.38 | (0.15- 0.97) | 0.044 |
| >150 m | 0.05 | (0.01-0.32) | 0.002 | 0.11 | (0.02-0.57) | 0.008 |
| **Distance water body class (**ref <1km**)** |  |  |  |  |  |  |
| 1 - 2 km | 0.10 | (0.02- 0.48) | 0.004 | - |  |  |
| >2 km | 0.23 | (0.04-1.35) | 0.104 | - |  |  |
|  |  |  |  |  |  |  |
| **Baseline intensity of infection (**ref: light infection**)** | 2.56 | (0.57- 11.5) | 0.219 | - |  |  |
| **Toilet in household** | 1.79 | (0.37- 8.67) | 0.471 | - |  |  |
| **Alpha** (Overdispersion parameter) |  |  |  | 21.1 | (16.1- 27.7) |  |

*IRR: Incidence Rate Ratio*

*aIRR: adjusted Incidence Rate Ratio*

*95%CI: 95% Confidence Intervals*
