# Supplementary material for: Impact of community piped water coverage on re-infection with urogenital schistosomiasis in rural South Africa
Source: eLife. 2020 Mar 17;9:e54012. doi: 10.7554/eLife.54012 (PMC7108860; doi:10.7554/eLife.54012)
Supplement: Supplementary file 2. — Model 1 presents results from a univariable negative binomial and Model 2 presents results from a multivariable negative binomial model (N = 125). [file elife-54012-supp2.docx]

**Supplementary File 2: Predictors of *S. haematobium* re-infection using data from the second follow up round only.** Model 1 presents results from a univariable negative binomial and Model 2 presents results from a multivariable negative binomial model (N=125).

|  | Model 1: Univariable  (N=125) | | | Model 2: Multivariable  (N=125) | | |
| --- | --- | --- | --- | --- | --- | --- |
| **Covariates** | **aIRR** | **95%CI** | **P-value** | **aIRR** | **95%CI** | **P-value** |
|  |  |  |  |  |  |  |
| **Female** | 0.20 | (0.02- 1.67) | 0.138 | 0.08 | (0.01- 0.47) | 0.005 |
|  |  |  |  |  |  |  |
| **community piped water coverage** | 0.93 | (0.87- 0.99) | 0.044 | 0.92 | (0.87- 0.97) | 0.002 |
|  |  |  |  |  |  |  |
| **Age at testing** | 0.80 | (0.52- 1.23) | 0.304 | 1.17 | (0.72- 1.89) | 0.529 |
| **Altitude Class (**ref <100 m**)** |  |  |  |  |  |  |
| >100 m | 0.20 | (0.02- 1.68) | 0.138 | 0.10 | (0.02- 0.44) | 0.002 |
| **Distance water body class (**ref <1km**)** |  |  |  |  |  |  |
| 1-2 km | 0.11 | (0.02- 0.70) | 0.019 | - |  |  |
| >2 km | 0.02 | (0.00- 0.12) | <0.001 | - |  |  |
|  |  |  |  |  |  |  |
| **Baseline intensity of infection (ref light infection)** | 2.64 | (0.43- 16.3) | 0.295 | - |  |  |
| **Alpha** (Overdispersion parameter) |  |  |  | 27.9 | (18.1- 42.8) |  |
