# Supplementary material for: Impact of community piped water coverage on re-infection with urogenital schistosomiasis in rural South Africa
Source: eLife. 2020 Mar 17;9:e54012. doi: 10.7554/eLife.54012 (PMC7108860; doi:10.7554/eLife.54012)
Supplement: Supplementary file 4. — Piped water coverage (exposure variable) was similar between participants who dropped out of the study and those that were enrolled and examined. Significantly higher dropouts were only observed among girls. Piped water coverage was derived from the Gaussian kernel density estimation of radius three kilometers. [file elife-54012-supp4.docx]

**Supplementary File 4:** Characteristics of participants who dropped out of the study at follow-up round 2. Piped water coverage (exposure variable) was similar between participants who dropped out of the study and those that were enrolled and examined. Significantly higher dropouts were only observed among girls. Piped water coverage was derived from the Gaussian kennel density estimation of radius 3 kilometers.

|  | |  | Attrition | | Piped water coverage in 2007  median % (Iqr) | |
| --- | --- | --- | --- | --- | --- | --- |
| Characteristic at baseline | | Infected at baseline  (N) | Dropouts at round 2 n(%) | P-values | subjects examined at round 2 | Dropouts at  round 2 |
| Sex | Female | 116 | 82 (70.7) | 0.006 ^a^ | 84.6 (65.0-97.2) | 85.2 (72.8-96.3) |
|  | Male | 217 | 120 (55.3) |  | 92.8 (73.8-98.1) | 92.4 (75.5-98.6) |
|  |  |  |  |  |  |  |
| Age category | <=10 | 57 | 28 (49.1) | 0.0909 ^b^ | 84.7 (67.5-98.4) | 80.3 (62.6-96.7) |
|  | 11 | 87 | 51 (58.6) |  | 92.5 (75.3-97.2) | 82.3 (73.6-97.1) |
|  | 12 | 92 | 63 (68.5) |  | 89.2 (64.3-97.7) | 90.3 (74.6-96.8) |
|  | ≥13 | 97 | 60 (61.9) |  | 93.9 (81.4-98.2) | 93.3 (78.6-98.3) |
|  |  |  |  |  |  |  |
| Grade | five | 185 | 107(57.8) | 0.238 ^a^ | 91.2 (67.7-97.5) | 87.5 (74.3-97.3) |
|  | six | 148 | 95 (64.2) |  | 94.3 (70.8-98.0) | 92.0 (73.6-97.4) |
|  |  |  |  |  |  |  |
| Altitude (Metres) | <50 | 29 | 15 (51.7) | 0.7903 ^b^ | 95.3 (91.5-99.2) | 82.0 (74.6-98.4) |
|  | 50-100 | 167 | 105 (62.9) |  | 82.1 (64.3-95.9) | 82.3 (69.2-96.3) |
|  | >100 | 137 | 82 (59.9) |  | 96.1 (81.4-98.9) | 95.1 (80.8-98.9) |
|  |  |  |  |  |  |  |
| Distance water body class | <1km | 112 | 66 (58.9) | 0.7655 ^b^ | 94.3 (66.8-98.1) | 93.6 (74.7-98.4) |
|  | 1-2km | 122 | 80 (65.6) |  | 91.2 (72.3-98.2) | 84.6 (72.3-97.9) |
|  | >2km | 99 | 56 (56.7) |  | 89.2 (77.6-97.3) | 85.1 (77.6-96.3) |
|  |  |  |  |  |  |  |
| Land cover class | Sparse Shrubland | 52 | 33 (63.5) | 0.609 ^a^ | 98.1 (89.8-99.1) | 97.9 (88.3-99.4) |
|  | Closed Shrubland | 183 | 115 (62.8) |  | 82.6 (67.9-96.8) | 82.9 (71.5-96.3) |
|  | Open Shrubland | 83 | 46 (55.4) |  | 94.3 (70.8-99.7) | 92.0 (76.2-96.9) |
|  | Thickett | 15 | 8 (53.3) |  | 91.9 (91.5-94.9) | 85.0 (73.9-96.9) |
|  |  |  |  |  |  |  |

*Iqr: interquartile range*

*^a^ refers to the p-value derived from the Chi-square analysis for nominal data and*

*^b^ refers to p-values derived from the Chi-square trend test*
