# Supplementary material for: Impact of community piped water coverage on re-infection with urogenital schistosomiasis in rural South Africa
Source: eLife. 2020 Mar 17;9:e54012. doi: 10.7554/eLife.54012 (PMC7108860; doi:10.7554/eLife.54012)
Supplement: Transparent reporting form [file elife-54012-transrepform.docx]

***eLife’s* transparent reporting form**

We encourage authors to provide detailed information *within their submission* to facilitate the interpretation and replication of experiments. Authors can upload supporting documentation to indicate the use of appropriate reporting guidelines for health-related research (see [EQUATOR Network](http://www.equator-network.org/%20)), life science research (see the [BioSharing Information Resource](https://biosharing.org/)), or the [ARRIVE guidelines](http://www.plosbiology.org/article/info:doi/10.1371/journal.pbio.1000412) for reporting work involving animal research. Where applicable, authors should refer to any relevant reporting standards documents in this form.

If you have any questions, please consult our Journal Policies and/or contact us: [editorial@elifesciences.org](mailto:editorial@elifesciences.org).

**Sample-size estimation**

- You should state whether an appropriate sample size was computed when the study was being designed
- You should state the statistical method of sample size computation and any required assumptions
- If no explicit power analysis was used, you should describe how you decided what sample (replicate) size (number) to use

Please outline where this information can be found within the submission (e.g., sections or figure legends), or explain why this information doesn’t apply to your submission:

In this study, all children who were infected with Schistosomiasis and treated at baseline were invited to participate in the follow-up re-infection surveys. In this regard we did not compute the sample size. We have discussed this limitation in the discussion section.

“A further limitation to this study is that we were powered to detect variations in infection prevalence at baseline as previously reported. The re-infection cohort inevitably created imbalances in sample sizes within variable categories.”

**Replicates**

- You should report how often each experiment was performed
- You should include a definition of biological versus technical replication
- The data obtained should be provided and sufficient information should be provided to indicate the number of independent biological and/or technical replicates
- If you encountered any outliers, you should describe how these were handled
- Criteria for exclusion/inclusion of data should be clearly stated
- High-throughput sequence data should be uploaded before submission, with a private link for reviewers provided (these are available from both GEO and ArrayExpress)

Please outline where this information can be found within the submission (e.g., sections or figure legends), or explain why this information doesn’t apply to your submission:

In the method section and the re-infection cohort subsection we have clearly presented information regarding the number of follow up rounds and the inclusion criteria. As follows:

“**Re-infection cohort design**

The baseline survey was conducted between May and July of 2007 whilst the two re-infection follow-up rounds were conducted between September and October of 2007 (Round 1), and between April and May of 2008 (Round 2). The re-infection cohort included children who had microscopically confirmed *S. haematobium* infection at baseline, received treatment for baseline infection and provided informed consent to participate in any of the follow-up rounds. Participants with confirmed infection at baseline or during the follow-up rounds were treated immediately at school. *S. haematobium* infection was determined using the rapid diagnostic tests (RDT) with confirmation using microscopy diagnosis. A single oral dose of 40 mg/kg body weight of praziquantel was administered to only infected children following recommended World Health Organization (WHO) guidelines on *S. haematobium* preventive chemotherapy [5, 6]. However, because of the time lag between laboratory testing and treatment, some children with a false negative RDT result missed treatment for baseline infection if they were absent from school. In addition, some parents/ guardians of children who were screened and treated for baseline infection refused consent for their children to participate in the follow-up rounds (Figure 1).

Therefore, the re-infection cohort was defined as children who had microscopically confirmed *S. haematobium* infection at baseline, were treated for baseline infection and consented to participate in at least one follow-up round. Eligibility for the second round of follow-up was subject to treatment at round one (for those found to be infected during screening) and consent at round two regardless of the infection status at round one, or children who were treated for baseline infection and consented for only round two of screening and treatment.

”

**Statistical reporting**

- Statistical analysis methods should be described and justified
- Raw data should be presented in figures whenever informative to do so (typically when N per group is less than 10)
- For each experiment, you should identify the statistical tests used, exact values of N, definitions of center, methods of multiple test correction, and dispersion and precision measures (e.g., mean, median, SD, SEM, confidence intervals; and, for the major substantive results, a measure of effect size (e.g., Pearson's r, Cohen's d)
- Report exact p-values wherever possible alongside the summary statistics and 95% confidence intervals. These should be reported for all key questions and not only when the p-value is less than 0.05.

Please outline where this information can be found within the submission (e.g., sections or figure legends), or explain why this information doesn’t apply to your submission:

We have described the statistical analysis methods in the data analysis section (page 10-12) of the manuscript. All effect sizes, exact p-values, 95% confidence intervals and the absolute numbers have been presented in Table 1and 2 and Supplementary Table 1 and Supplementary table 2

(For large datasets, or papers with a very large number of statistical tests, you may upload a single table file with tests, Ns, etc., with reference to sections in the manuscript.)

**Group allocation**

- Indicate how samples were allocated into experimental groups (in the case of clinical studies, please specify allocation to treatment method); if randomization was used, please also state if restricted randomization was applied
- Indicate if masking was used during group allocation, data collection and/or data analysis

Please outline where this information can be found within the submission (e.g., sections or figure legends), or explain why this information doesn’t apply to your submission:

This information is not required given that our study involved a cohort of children who were treated at baseline and followed up to assess reinfection status at two timepoints

**Additional data files (“source data”)**

- We encourage you to upload relevant additional data files, such as numerical data that are represented as a graph in a figure, or as a summary table
- Where provided, these should be in the most useful format, and they can be uploaded as “Source data” files linked to a main figure or table
- Include model definition files including the full list of parameters used
- Include code used for data analysis (e.g., R, MatLab)
- Avoid stating that data files are “available upon request”

Please indicate the figures or tables for which source data files have been provided:

Not applicable
